# Genetic diversity, natural selection and haplotype grouping of *Plasmodium vivax* Duffy-binding protein genes from eastern and western Myanmar borders

**DOI:** 10.1186/s13071-019-3803-2

**Published:** 2019-11-20

**Authors:** Yubing Hu, Lin Wang, Huguette Gaelle Ngassa Mbenda, Myat Thu Soe, Chunyun Yu, Hui Feng, Myat Phone Kyaw, Liwang Cui, Xiaotong Zhu, Yaming Cao

**Affiliations:** 1 Department of Immunology, College of Basic Medical Science, China Medical University, Shenyang, 110122 Liaoning China; 2 Department of Internal Medicine, Morsani College of Medicine, University of South Florida, 3720 Spectrum Boulevard, Suite 304, Tampa, FL 33612 USA; 3 Myanmar Health Network Organization, Yangon, Myanmar

**Keywords:** *Plasmodium vivax*, Duffy-binding protein, Genetic diversity, Myanmar border

## Abstract

**Background:**

Merozoite proteins of the malaria parasites involved in the invasion of red blood cells are selected by host immunity and their diversity is greatly influenced by changes in malaria epidemiology. In the Greater Mekong Subregion (GMS), malaria transmission is concentrated along the international borders and there have been major changes in malaria epidemiology with *Plasmodium vivax* becoming the dominant species in many regions. Here, we aimed to evaluate the genetic diversity of *P. vivax Duffy-binding protein* gene domain II (*pvdbp-*II) in isolates from the eastern and western borders of Myanmar, and compared it with that from global *P. vivax* populations.

**Methods:**

*pvdbp*-II sequences were obtained from 85 and 82 clinical *P. vivax* isolates from the eastern and western Myanmar borders, respectively. In addition, 504 *pvdbp-*II sequences from nine *P. vivax* populations of the world were retrieved from GenBank and used for comparative analysis of genetic diversity, recombination and population structure of the parasite population.

**Results:**

The nucleotide diversity of the *pvdbp*-II sequences from the Myanmar border parasite isolates was not uniform, with the highest diversity located between nucleotides 1078 and 1332. Western Myanmar isolates had a unique R391C mutation. Evidence of positive natural selection was detected in *pvdbp*-II gene in *P. vivax* isolates from the eastern Myanmar area. *P. vivax* parasite populations in the GMS, including those from the eastern, western, and central Myanmar as well as Thailand showed low-level genetic differentiation (*F*_ST_, 0.000–0.099). Population genetic structure analysis of the *pvdbp*-II sequences showed a division of the GMS populations into four genetic clusters. A total of 60 PvDBP*-*II haplotypes were identified in 210 sequences from the GMS populations. Among the epitopes in PvDBP*-*II, high genetic diversity was found in epitopes 45 (379-SIFGT(D/G)(E/K)(K/N)AQQ(R/H)(R/C)KQ-393, π = 0.029) and Ia (416-G(N/K)F(I/M)WICK(L/I)-424], Ib [482-KSYD(Q/E)WITR-490, π = 0.028) in *P. vivax* populations from the eastern and western borders of Myanmar.

**Conclusions:**

The *pvdbp*-II gene is genetically diverse in the eastern and western Myanmar border *P. vivax* populations. Positive natural selection and recombination occurred in *pvdbp*-II gene. Low-level genetic differentiation was identified, suggesting extensive gene flow of the *P. vivax* populations in the GMS. These results can help understand the evolution of the *P. vivax* populations in the course of regional malaria elimination and guide the design of PvDBP-II-based vaccine.
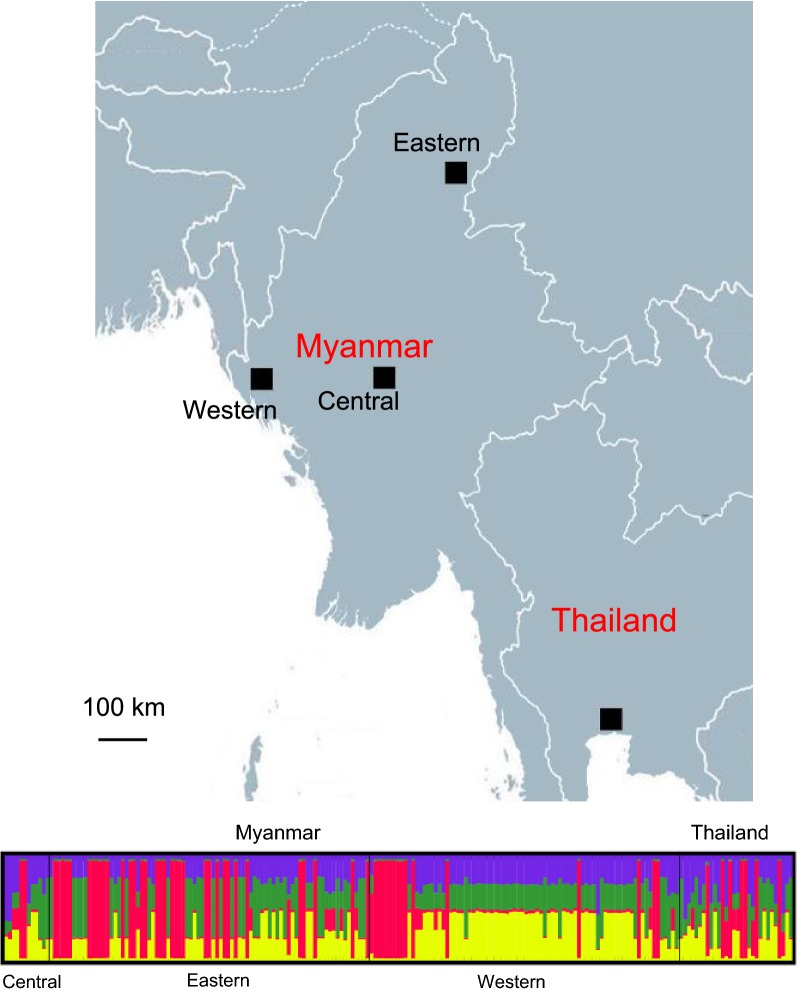

## Background

Malaria is a major public health concern in the Great Mekong Subregion (GMS) in Asia [1]. In this region, Myanmar has the heaviest malaria burden and contributes to over 50% of the malaria cases in the GMS [2–4]. In recent years, intensified malaria control efforts have resulted in substantial improvement of the malaria situation in most GMS countries. One noticeable change in malaria epidemiology is the emerging dominant status of *P. vivax* in most endemic areas of the GMS. The formation of liver hypnozoites in *P. vivax* patients responsible for relapses requires radical treatment that targets both asexual blood stages and liver-stage hypnozoites. Currently, chloroquine–primaquine (CQ–PQ) is the frontline therapy for *P. vivax* in all GMS countries [5]. However, degraded efficacy of CQ–PQ for *P. vivax* treatment has been notified in several sites in Myanmar [5–8]. This might be among the causes responsible for the recently increased *P. vivax* incidence in northeastern Myanmar bordering China’s Yunnan province [5, 9]. The development of an effective malaria vaccine is considered important for integrated malaria control. However, one of the main obstacles for a successful vaccine design to enable global protection is the extensive genetic diversity of vaccine candidate genes [10]. In this regard, to design an effective malaria vaccine, it is essential to determine the genetic diversity of the vaccine targets in parasite populations from different malaria-endemic areas.

Blood-stage replication of malaria parasites is the cause of clinical malaria, and vaccines targeting blood-stage parasites are designed to alleviate the clinical symptoms. The formation of tight junction between the *Plasmodium* merozoite and the host red blood cell (RBC) is central to the invasion process of the parasite. This process in *P. vivax* involves the Duffy-binding protein (PvDBP), a microneme protein, and the Duffy antigen receptor for chemokines (DARC) on the RBC membrane [11, 12]. Although *P. vivax* could infect DARC-negative individuals, these cases are rare; no alternative ligands for *P. vivax* binding to reticulocytes have been identified yet [13–16]. The presence of naturally-acquired antibodies to PvDBP in sera of residents from endemic area that block RBC invasion suggests that this protein is a potential target for effective antibody response [17–19]. Naturally-acquired binding-inhibitory antibodies directed against PvDBP contained both strain-specific and strain-transcending components and were associated with a reduced risk of clinical *P. vivax* malaria. Furthermore, these antibodies against PvDBP could block erythrocyte binding of PvDBP domain II (PvDBP-II) and inhibit merozoite invasion of erythrocytes [20–24], which justifies PvDBP as one of the most promising targets for blood-stage vaccine development against *P. vivax*.

PvDBP is a protein of 140 kDa and is divided into seven different regions (regions I–VII). The central binding motif necessary for DARC adhesion of PvDBP is mapped to a 170 amino-acid stretch located in the N-terminal cysteine-rich region (PvDBP-II, amino acids 291–460) [25]. *Pvdbp*-II encoding this protein domain also shows the highest degree of genetic diversity as compared to the rest of the *pvdbp* sequence. Although the cysteine residues are conserved in *P. vivax* populations, the remaining amino acids of PvDBP-II are highly polymorphic in *P. vivax* field isolates from endemic areas such as Brazil, Colombia, South Korea, Thailand and Myanmar, suggesting that this region is under positive natural selection [3, 26–29]. It has been reported that these polymorphic residues within the PvDBP-II region do not alter its capacity to bind DARC, but some of them affect the immune recognition of PvDBP, suggesting that genetic diversity may be responsible for immune evasion [30, 31]. Consequently, the polymorphic nature of *pvdbp-II* represents a major limitation in successful design of a protective vaccine. Therefore, understanding the nature and genetic polymorphism in *pvdbp*-II among *P. vivax* isolates from different geographic areas is important for the rational design of vaccines against *vivax* malaria.

In this study, the *pvdbp*-II sequences of *P. vivax* isolates from eastern and western Myanmar borders were analyzed for genetic diversity, natural selection, recombination, haplotype prevalence and population differentiation, and were compared with the global *pvdbp*-II sequences. Whereas limited polymorphism of *pvdbp* existed in the field *P. vivax* isolates from eastern and western Myanmar, the *pvdbp*-II region was found to be under natural selection. Besides, the observed low-level genetic differentiation among different *P. vivax* populations in the GMS, based on the *pvdbp*-II sequence analysis, supports a general design of an effective vaccine to cover these endemic areas.

## Methods

### Blood sample collection

**Fig. 1:**
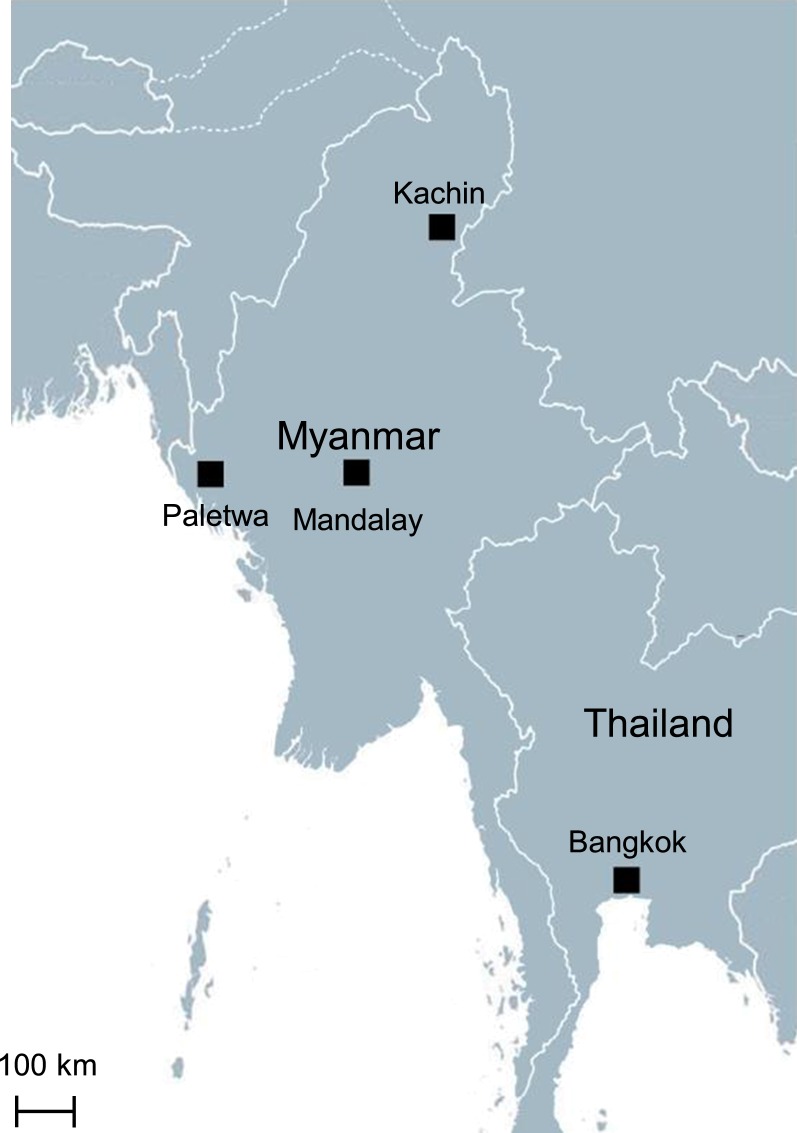
Map showing the distribution of sampling sites including Kachin State (eastern Myanmar), Mandalay Division (central Myanmar), Chin State (western Myanmar), and Thailand

A total of 163 and 95 blood samples were collected from patients with acute *P. vivax* malaria attending clinics in an eastern Myanmar border township (Laiza, Kachin State) in 2016 and a western Myanmar border township (Paletwa, Chin State) in 2017, respectively (Fig. 1). *Plasmodium vivax* infection was confirmed by microscopic examination of thin and thick blood smears. After obtaining written informed consent from participants and guardians in case of minors, finger-prick blood (~ 100 µl) was collected on Whatman 3M filters (Whatman, Shanghai, China), dried and individually stored at − 20 °C for subsequent use. This study was approved by the ethics committees from the Department of Medical Research, Myanmar Ministry of Health and Sports, the China Medical University, and the University of South Florida.

### PCR, cloning and sequencing of the *pvdbp*-II gene

Genomic DNA was extracted from dried blood spots using a QIAamp DNA mini kit (Qiagen, Hilden, Germany) according to the manufacturer’s protocol. The *pvdbp*-II sequence corresponding to nucleotides 715–1704 bp (amino acid positions 239–568 aa) in the Salvador I (Sal I) reference sequence (GenBank: M37514) was amplified by PCR using primers *pvdbp*-II-F (5′-ACC ACG ATC TCT AGT GCT ATT ATA-3′) and *pvdbp*-II-R (5′-ATT TGT CAC AAC TTC CTG AGT ATT-3′). PCR reactions included 1× KOD-Plus-Neo buffer, 200 μM dNTPs, 1 mM MgSO_4_, 250 nM of each primer, 0.4 units of KOD-Plus-Neo DNA polymerase (Toyobo, Shanghai, China), and 1.0 μl genomic DNA template. The PCR was performed using the following conditions: 95 °C for 5 min, 44 cycles at 95 °C for 30 s, 55 °C for 30 s, and 68 °C for 60 s, followed by extension at 68 °C for 5 min. The PCR products were analyzed by 1.2% agarose gel, purified with TaKaRa MiniBEST DNA Fragment Purification Kit (TaKaRa, Dalian, China), ligated into a pMD18-T plasmid vector (TaKaRa), and transformed into *Escherichia coli* DH5α competent cells (TaKaRa). Colony PCR was performed with the primers M13(-20)F (5′-TGT AAA ACG ACG GCC AGT-3′) and M13R (5′-CAG GAA ACA GCT ATG ACC-3′) to identify positive clones with the correct inserts. The colony PCR products having the correct DNA fragment were sequenced using the primers *pvdbp*-II-F and *pvdbp*-II-R at a commercial laboratory (Beijing Genomics Institute, Beijing, China). To verify the sequencing accuracy, at least two clones from each isolate were sequenced in both DNA strands. Nucleotide sequences are deposited in GenBank under accession numbers MN233407-MN233488 for the western Myanmar isolates and MN233489-MN233573 for the eastern Myanmar isolates.

### Genetic diversity and natural selection

To estimate within-population diversity, the DNA sequence polymorphism analysis was performed on *pvdbp*-II sequences from 82 western 85 eastern Myanmar border parasite isolates, respectively. Sequence alignment was performed using the CLUSTAL W program in MEGA7.0 [32] and nucleotide diversity was analyzed using the DnaSP5.10.01 software [33]. To compare the genetic diversity of *pvdbp*-II gene with *pvdbp*-II sequences in global *P. vivax* isolates, we retrieved from GenBank *pvdbp*-II sequences from Asia: India (*n* = 100, FJ491142.1-FJ491241.1), Iran (*n* = 63, EU860428.1-EU860436.1, KF318358.1, KF318359.1, KF791921.1-KF791926.1) [17, 34, 35], central Myanmar (*n* = 12, JN255576.1-JN255587.1) [3], South Korea (*n* = 13, JN989472.1-JN989484.1) [28], Sri Lanka (*n* = 100, GU143914-GU143949, GU143950-GU143973, GU143974-GU144013) [36], and Thailand (*n* = 30, EF379127.1-EF379132.1, EF379134.1, EF368159.1-EF368180.1, EF219451.1) [29]; South America: Brazil (*n* = 122, EU812839-EU812960) [26] and Colombia (*n* = 17, U50575-U50590) [27]; and Oceania: Papua New Guinea (*n* = 96, AF289480-AF289483, AF289635-AF289653, AF469515-AF469602) [37]. The number of haplotypes (*H*), haplotype diversity (*H*d), nucleotide diversity (π), number of segregating sites (*S*), the total number of mutations (*η*), the average number of nucleotide differences (*k*) and the corresponding standard deviation for the *pvdbp*-II fragments were calculated using the DnaSP [33]. Additionally, the π value was also estimated on a sliding window of 90 bases with a step size of 3 bp. The rates of synonymous (*d*_S_) and non-synonymous (*d*_N_) mutations were estimated and compared by the *Z-*test (*P* < 0.05) in MEGA 7.0 using the Nei & Gojobori’s method with Jukes & Cantor correction and 1000 bootstrap replications [38]. Under purifying selection, *d*_N_ will be less than *d*_S_ (*d*_N_/*d*_S_ < 1), whereas under positive selection, *d*_N_ will exceed *d*_S_ (*d*_N_/*d*_S_ > 1) [38]. The Tajima’s *D*-test [39], Fu and Li’s D^*^ and F^*^ tests [40] were used to test departures from the neutral theory of evolution, with the assumption that the population size was constant [41]. Sliding window plots with a window size of 90 bp and a step size of 3 bp were also performed for Tajima’s *D* and Fu and Li’s D* and F* tests implemented in DnaSP [33].

### Recombination and linkage disequilibrium (LD)

The recombination parameter (C), which includes the effective population size and probability of recombination between adjacent nucleotides per generation, and the minimum number of recombination events (Rm) were measured using DnaSP [42]. LD between different polymorphic sites was computed in terms of the R^2^ index using DnaSP for the eastern and western Myanmar border isolates.

### Genetic differentiation, haplotype network, and population genetic structure analysis

The genetic differences in populations was investigated evaluating the rate of fixation (*F*_ST_) by analysis of molecular variance (AMOVA) implemented in ARLEQUIN v3.5.2.2 software [43]. The Median-Joining method in NETWORK v5.0.0.3 [44] was used to establish genealogical relationship among the global PvDBP-II haplotypes. STRUCTURE 2.3.4 was used to define the genetic structure of *P. vivax* parasite populations from the GMS based on the *pvdbp*-II [45]. The Bayesian approach was employed to identify the optimum number of clusters (K). All sample data were run for values K = 2–9 using the admixture ancestry model. Each run was implemented with a ‘burn-in’ period of 50,000 iterations and 100,000 Markov Chain Monte Carlo (MCMC) replications. The most likely number K in the data was estimated by calculating Delta K-values and identifying the K-value that maximizes the log probability of data, |ln’’(K)|. The most probable K-value was then calculated according to Evanno’s method by using the webpage interface STRUCTURE Harvester (http://taylor0.biology.ucla.edu/structureHarvester/). Distruct 1.1 was used to graphically display results produced by the STRUCTURE software [46].

### Analysis of polymorphism associated with B- and T-cell epitopes

To evaluate the possibility that diversity in PvDBP-II within the eastern and western Myanmar border isolates may have arisen from host’s immune pressure, the genetic diversity in predicted or known B- and T-cell epitopes and MHC-binding regions in PvDBP-II was examined [26, 30]. Polymorphism of each region was analyzed by using DnaSP as described above.

## Results

### *Pvdbp*-II genetic diversity in the Myanmar border *P. vivax* populations

**Fig. 2:**
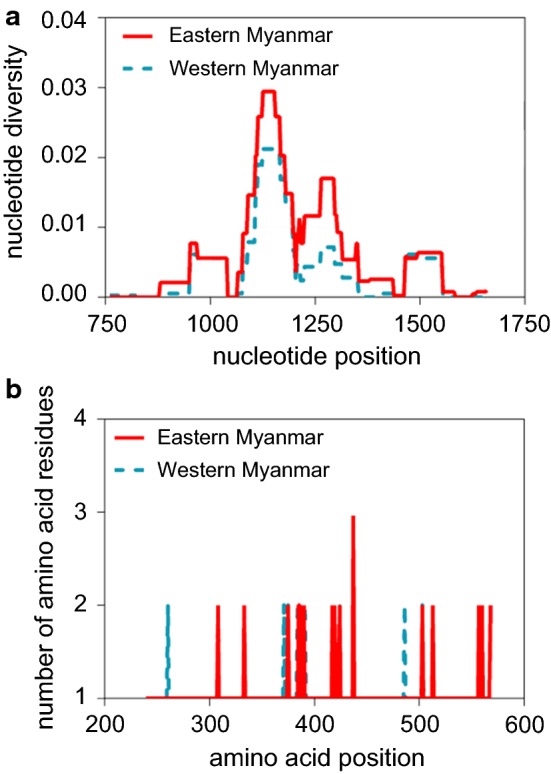
Sliding window plots of nucleotide diversity (**a**) and amino acid polymorphism (**b**) of *pvdbp*-II in eastern and western Myanmar populations. The analysis included 85 and 82 sequences from the eastern and western Myanmar borders, respectively. Nucleotide and amino acid positions are after the Salvador I strain

Out of the 258 *P. vivax* isolates collected from the eastern (*n* = 95) and western (*n* = 163) Myanmar borders, the 990 bp *pvdbp*-II gene fragment covering codons 239–568 was successfully amplified and sequenced in 85 and 82 samples, respectively. The 85 eastern border samples had 21 single nucleotide polymorphisms (SNPs), of which 4 are synonymous and 17 non-synonymous (Table 1). Among them, 20 are segregating sites, and 15 are parsimony informative. The 82 western border samples contained 16 SNPs, of which 15 are non-synonymous. All 16 SNPs are segregating sites, and 14 are parsimony informative. The overall haplotype diversity for the two sample sets was similar at 0.850 and 0.930 for the eastern and western border populations, respectively (Table 1). The observed pairwise nucleotide diversity for eastern and western Myanmar isolates is 0.006 and 0.004, respectively (Table 1). The highest peaks of nucleotide diversity within the *pvdbp*-II region were identified between nucleotide positions 1078–1332 (codons 360–444) (Fig. 2a). Meanwhile, 40% of the polymorphic amino acid sites were also accumulated within this segment (Fig. 2b). Sliding window plots of nucleotide diversity revealed π value ranging from 0 to 0.029 for the eastern isolates and from 0 to 0.021 for the western isolates, respectively (Fig. 2). These results indicated that the central segment for codons 360–444 of the *pvdbp*-II gene is more polymorphic than the rest of the *pvdbp* domain II.

**Table 1:** Nucleotide diversity of *pvdbp*-II in Myanmar *P. vivax* isolates

Region	n	*S*	*η*	*NS*	*SP*	*k*	π ± SD	H	*Hd* ± SD
Eastern Myanmar	85	20	21	17	4	5.784	0.006 ± 0.000	16	0.850 ± 0.021
Western Myanmar	82	16	16	15	1	3.694	0.004 ± 0.000	25	0.930 ± 0.013

*Note*: The total sequenced region includes codons 239 to 568

*Abbreviations*: *S*, number of polymorphic (segregating) sites; *η*, the total number of mutations; *NS*, number of non-synonymous polymorphisms, *SP*, number of synonymous polymorphisms; *k*, the average number of nucleotide differences, π, pairwise nucleotide diversity; H, number of haplotypes; *Hd*, haplotype diversity

Most of the PvDBP-II amino acid changes were found in high frequencies (> 50%) in both populations, including L333F (eastern, 77.7%; western, 85.4%), D384G (eastern, 100%; western, 92.7%), R390H (eastern, 80.0%; western, 92.7%), N417K (eastern, 63.5%; western, 87.8%), and L424I (eastern, 77.7%; western, 85.4%) (Table 2). The variant E385K (eastern, 50.6%; western, 26.8%), K386N (eastern, 50.6%; western, 26.8%) and W437R (eastern, 62.4%; western, 2.4%) were observed with higher frequency in eastern Myanmar than those from western Myanmar isolates. It is interesting to note that within the commonly evaluated 299–503 aa region of PvDBP-II, 13 of the 14 non-synonymous changes in eastern Myanmar border isolates and western Myanmar isolates were previously reported, whereas the R391C (1.22 %) change was unique to western Myanmar isolates.

**Table 2:** Nucleotide and amino acid changes of *pvdbp*-II among Myanmar isolates

Nucleotide position	Codon	Wild-type	Mutant	Amino acid substitution	Frequency (%)	
					Eastern Myanmar (*n* = 85)	Western Myanmar (*n* = 82)
778	260	AAA	GAA	K - E	0	1.22
924	308	AGG	AGT	R - S	10.59	2.44
997	333	CTT	TTT	L - F	50.59	50.00
1111	371	AAA	GAA	K - E	20.00	2.44
1123	375	AAT	GAT	N - D	44.71	21.95
1134	**378**	**CGC**	**CGT**	**R - R**	**42.35**	**19.51**
1151	384	GAT	GGT	D - G	100.00	92.68
1153	385	GAA	AAA	E - K	50.59	35.37
1158	386	AAG	AAT	K - N	50.59	26.83
1169	390	CGT	CAT	R - H	80.00	92.68
1251	417	AAT	AAA	N - K	63.53	87.80
1257	419	ATA	ATG	I - M	12.94	0
1270	424	TTA	ATA	L - I	77.65	85.37
1309	437	TGG	CGG	W - R	62.35	2.44
1392	**464**	**ATC**	**ATA**	**I - I**	**11.76**	**0**
1425	**475**	**CCA**	**CCC**	**P - P**	**1.18**	**0**
1456	486	CAA	GAA	Q - E	0	2.44
1508	503	ATA	AAA	I - K	47.06	48.78
1538	513	ACG	AAG	T - K	3.53	0
1669	557	GCT	ACT	A - T	1.18	0
1671	**557**	**GCT**	**GCC**	**A - A**	**1.18**	**0**
1679	560	AAT	ATT	N - I	1.18	0
1703	568	GTC	GGG	V - G	1.18	0

*Notes*: Number of the amino acid residues is according to Sal I sequence. Text highlighted in bold indicates non-synonymous mutations

### Evidence of natural selection

**Fig. 3:**
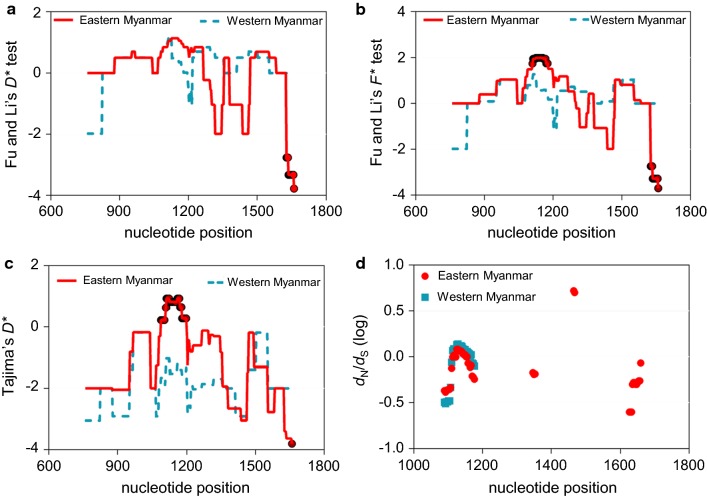
Neutrality tests of *pvdbp*-II sequences from the eastern and western Myanmar isolates. Sliding window plots of Fu and Li’s D* (**a**), Fu and Li’s F* (**b**), and Tajima’s *D* (**c**) for the *pvdbp*-II sequence. Sites significantly departed from neutrality (*P *< 0.05, two-tailed) are indicated with circle symbols on the line. **d** Sliding window plots of *d*_N_/*d*_S_ ratio for the *pvdbp*-II sequences. Nucleotide positions are after the Sal I sequence. Window length is 90 bp and step size is 3 bp

The neutrality tests (Tajima’s *D*, and Fu and Li’s D*, and F*) did not identify significant deviation from zero in *pvdbp*-II in both border populations (Table 3). However, by zooming in specific regions via the sliding window analysis, both Fu and Li’s D* and F* tests detected significant negative values for the 1626–1659 bp region in the eastern Myanmar population (Fu and Li’s D* test: − 3.777, *P* < 0.05; Fu and Li’s F* test: − 3.694, *P* < 0.05), whereas Fu and Li’s F* test detected a significant positive value for the 1110–1176 bp region (1.987, *P* < 0.05). In addition, a significant positive Tajima’s *D*-value was found in the 1089–1197 bp region from the eastern border isolates (2.810, *P* < 0.01). All the three neutrality tests identified similar patterns in the western Myanmar population, albeit the *P*-values did not reach statistical significance (*P* > 0.05) (Fig. 3a–c).

**Table 3:** Neutrality tests of *pvdbp*-II among Myanmar *P. vivax* isolates

Region	n	*d*_N_ ± SE	*d*_S_ ± SE	*d*_N_/*d*_S_	D	D*	F*
Eastern Myanmar	85	0.006 ± 0.002	0.004 ± 0.003	1.708	1.330	− 0.841	− 0.045
Western Myanmar	82	0.005 ± 0.001	0.001 ± 0.001)	2.712^*^	0.429	0.623	0.659

*Abbreviations*: *d*_N_/*d*_S_, the ratio of non-synonymous to synonymous mutations; D, Tajima’s *D* test; D*, Fu and Li’s D* value; F*, Fu and Li’s F* value; SE, standard error

*P < 0.05

To determine whether selection has played any role in the evolution of *pvdbp*-II in the two parasite populations, we compared the *d*_N_ and *d*_S_ values (Table 3). In both populations, *d*_N_/*d*_S_ > 1 was detected, but Fisher’s exact *Z*-test only showed significance for the western Myanmar isolates (*P* = 0.033). Sliding window plots of *d*_N_/*d*_S_ for the *pvdbp*-II sequences showed a similar pattern with a significant excess of *d*_N_ over *d*_S_ detected at the 1116–1164 bp region (codons 372–388) in the western Myanmar population (Fig. 3d).

### Recombination

**Fig. 4:**
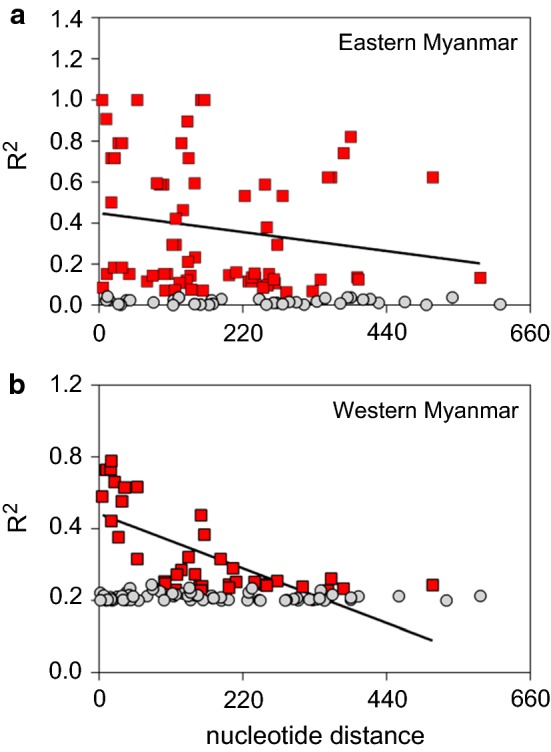
Linkage disequilibrium plots of R^2^ for *pvdbp*-II gene from eastern (**a**) and western (**b**) Myanmar isolates. Sites with significant LD (*P *<0.05) as detected by Fisher’s exact test are shown as red squares, whereas all others are shown as grey dots. Trace lines represent the regression lines

In the global *P. vivax* populations except that from South Korea, which had recent vivax malaria resurgence, the minimum numbers of recombination events between adjacent polymorphic sites (Rm) were ≥ 5 (Table 4). The LD index R^2^ also declined across the analyzed region in eastern and western Myanmar populations, suggesting that intragenic recombination may also be a possible factor contributing to the increased diversity of *pvdbp*-II gene (Fig. 4). It is also noteworthy that the R^2^ value decreased much more rapidly in the western Myanmar parasites, suggesting a much large parasite population and higher level of intragenic recombination in western Myanmar.

**Table 4:** Estimates of recombination events in *pvdbp-*II in global *P. vivax* populations

Locality (*n*)	R^a^	R^b^	Rm
Eastern Myanmar (*n* = 85)	0.011	7.3	5
Western Myanmar (*n* = 82)	0.021	13.9	7
India (*n* = 100)	0.024	16.1	10
Iran (*n* = 63)	0.014	9.4	5
Central Myanmar (*n* = 11)	0.191	128	7
South Korea (*n* = 13)	0.074	49.7	1
Sri Lanka (*n* = 100)	0.021	13.8	9
Thailand (*n* = 30)	0.152	102	7
Brazil (*n* = 122)	0.041	27.8	6
Colombia (*n* = 16)	0.071	47.9	6
PNG (*n* = 96)	0.017	11.4	8

*Note*: The analyzed region includes codons 292–516

*Abbreviations*: R^a^, recombinant parameter between adjacent sites; R^b^, recombinant parameter for the whole gene; Rm, minimum number of recombination events; n, total collected

### Population differentiation

*F*_ST_ values were calculated to assess the genetic differentiation of *pvdbp*-II among the global *P. vivax* populations. Though separated by more than 400 km, the eastern and western Myanmar *P. vivax* population had little genetic differentiation with an *F*_ST_ value of 0.067 (Table 5). Similarly, all *P. vivax* populations from the GMS had little genetic differentiation, whereas high levels of genetic differentiation were detected between the GMS populations and Asian Pacific population (PNG) and the South American population (Colombia).

**Table 5:** Genetic differentiation (*F*_ST_) of the *pvdbp-*II among 11 geographical populations

Locality (*n*)	Eastern Myanmar	Western Myanmar	Central Myanmar	India	Iran	South Korea	Sri Lanka	Thailand	Brazil	Colombia
Eastern Myanmar (*n* = 85)		0.001	0.151	0.000	0.000	0.002	0.000	0.057	0.000	0.000
Western Myanmar (*n* = 82)	0.067		0.011	0.000	0.000	0.000	0.000	0.000	0.000	0.000
Central Myanmar (*n* = 12)	0.033	0.096		0.026	0.000	0.003	0.009	0.877	0.020	0.000
India (*n* = 100)	0.125	0.181	0.066		0.000	0.005	0.019	0.001	0.040	0.000
Iran (*n* = 63)	0.225	0.285	0.113	0.045		0.000	0.000	0.000	0.019	0.000
South Korea (*n* = 13)	0.187	0.212	0.155	0.096	0.172		0.014	0.001	0.002	0.000
Sri Lanka (*n* = 100)	0.150	0.204	0.096	0.018	0.055	0.085		0.000	0.003	0.000
Thailand (*n* = 30)	0.031	0.099	0.000	0.075	0.141	0.146	0.010		0.000	0.000
Brazil (*n* = 122)	0.138	0.180	0.073	0.013	0.022	0.112	0.028	0.083		0.000
Colombia (*n* = 16)	0.328	0.431	0.241	0.199	0.185	0.337	0.203	0.237	0.176	
PNG (*n* = 96)	0.261	0.310	0.197	0.169	0.176	0.213	0.171	0.179	0.168	0.239

*Notes*: The nucleotide positions 784–1458 of *pvdbp*-II were used for population differentiation analysis. *F*_ST_ values are shown below the diagonal and *P*-values are shown above the diagonal

*Abbreviation*: n, total collected

### Relationship of parasites from the GMS

**Fig. 5:**
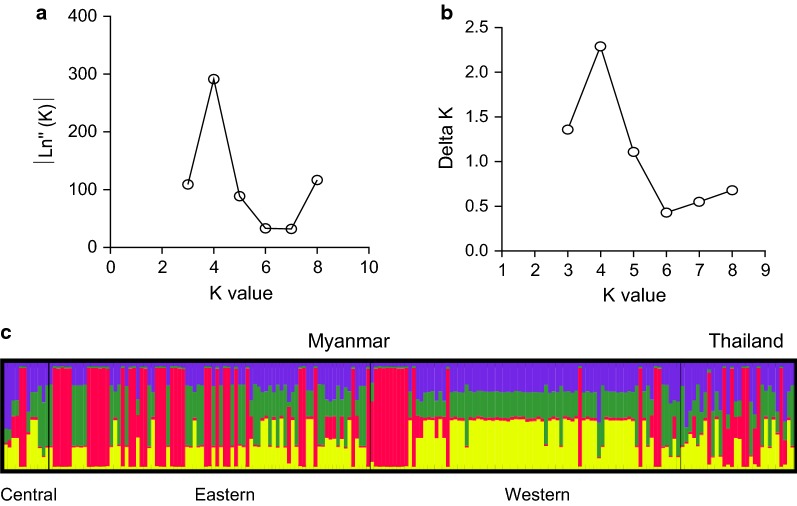
STRUCTURE analysis of PvDBP-II haplotypes. **a** |Ln’’(K)| plot. **b** Delta K Plot. **c** Clustering patterns of the PvDBP-II haplotypes (K = 4)

To identify the population structure of the GMS parasite populations, STRUCTURE analysis was performed using parasite populations from the eastern and western borders of Myanmar, central Myanmar (from the Mandalay region) and Thailand. Analysis of the |Ln’’(K)| curve, Delta K plot and the distribution of clusters amongst haplotypes indicated that the haplotypes were optimally grouped into four clusters (K = 4; Fig. 5). All four parasite populations from the GMS had admixed haplotypes, with the most common haplotypes found with a percentage of 79.1%, 76.1%, 81.2%, and 70.1% in western Myanmar border, eastern Myanmar border, central Myanmar and Thailand populations, respectively.

**Fig. 6:**
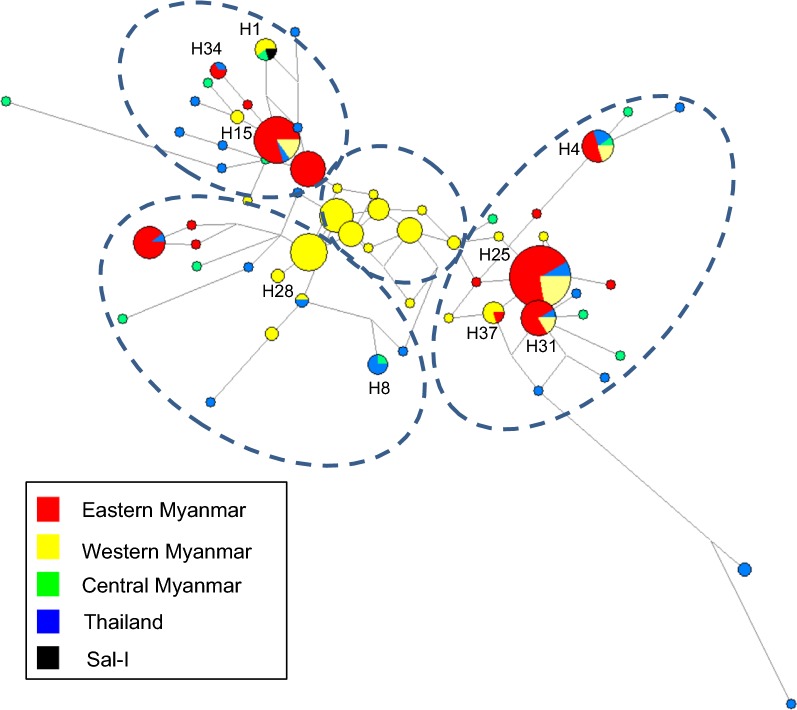
Network analysis of PvDBP-II haplotypes. Haplotypes composed of 224 amino acids were analyzed using the Median-Joining algorithm implemented in Network version 5.0.0.3 software. Pies represent the haplotypes and lines indicate connections between them. The size of each pie indicates the frequency of a particular haplotype. Colors of each pie indicate different countries. Four clusters are highlighted with dashed circles

A haplotype network was constructed to establish the relationships among the *pvdbp*-II haplotypes from the four GMS *P. vivax* populations mentioned above. A total of 60 haplotypes were identified from 210 *pvdbp*-II sequences with haplotype prevalence ranging from 0.48 to 17.14%, of which 65% was represented by single parasite isolates. These haplotypes could be roughly grouped into four clusters (Fig. 6). Parasites with the Sal I reference haplotype (H1) had a 2.3% prevalence, and it was only detected in the western and central Myanmar parasite populations. Haplotype 4 (H4) was shared among all GMS populations, with an overall prevalence of 4.8%. Haplotypes 8, 28, 34, and 37 were shared between two of the four populations, whereas Haplotypes 15, 25, and 31 were shared among the eastern Myanmar, western Myanmar and Thai populations. Of these haplotypes, H25 has an observed frequency of 17.14%, and it is the dominant haplotype of the eastern Myanmar parasite population with a frequency of 29.41% (Fig. 6).

### Polymorphisms associated with B- and T-cell epitopes

To determine whether there is a link between positive natural selection and host immune pressure, we examined the genetic polymorphism in identified or predicted B- and T-cell epitopes and MHC-binding regions [26, 30]. All the predicted B- and T-cell epitopes and MHC binding regions were polymorphic, and had *d*_N_ > *d*_S_ (Table 6). Meanwhile, significant *P*-value of *d*_N_ > *d*_S_ was detected in epitopes 45 (*P* = 0.023), 48 (*P* = 0.028) and Ia (*P* = 0.036) of PvDBP-II protein by *Z*-test (Table 6). Particularly, high-level nucleotide diversity was found in epitopes 45 (π = 0.029) and Ia (π = 0.028), which contained polymorphic residues at 339, 340, 341, 345, and 346, as well as 372, 374, and 379, respectively. Though the Tajima’s *D*-values for the two epitopes were all positive, they were not significant. These results are consistent with the hypothesis that natural selection acts on epitopes in PvDBP-II and is responsible for the observed diversity of *pvdbp*-II [3, 26, 36].

**Table 6:** Polymorphisms observed in each *pvdbp-*II epitope sequence

Epitope name	Epitope	*S*	*M*	*H*	*Hd* ± SD	π ± SD	*d*_N_-*d*_S_	Tajima’s *D*
5	T/B	1	1	2	0.124 ± 0.033	0.003 ± 0.007	0.003	− 0.308
16	T/B	1	1	2	0.503 ± 0.000	0.012 ± 0.000	0.016	1.942
18	B	1	1	2	0.503 ± 0.000	0.011 ± 0.000	0.014	1.942
20	T/B	1	1	2	0.503 ± 0.000	0.015 ± 0.000	0.019	1.942
45	B	5	5	8	0.674 ± 0.024	0.029 ± 0.001	0.036*	0.966
48	B	2	2	3	0.266 ± 0.039	0.007 ± 0.001	0.008*	− 0.333
66	T	1	1	2	0.273 ± 0.039	0.006 ± 0.001	0.008	0.576
Ia	MHC class I	3	3	4	0.491 ± 0.041	0.028 ± 0.003	0.034*	0.757
Ib	MHC class I	1	1	2	0.024 ± 0.016	0.001 ± 0.001	0.001	− 0.901
Ic	MHC class I	1	1	2	0.502 ± 0.005	0.012 ± 0.000	0.015	1.937
IIa	MHC class II	1	1	2	0.373 ± 0.034	0.012 ± 0.001	0.016	1.169
IIb	MHC class II	2	2	3	0.375 ± 0.042	0.015 ± 0.002	0.018	0.181

*Notes*: Epitope sequences are: 5 [299-VNNTDTNFH(R/S)DITFR-313]; 16 [321-LIYDAAVEGDLL(L/F)KL-335]; 18 [325-AAVEGDLL(L/F)KLNNYR-339]; 20 [329-GDLL(L/F)KLNNYRYNKD-343]; 45 [379-SIFGT(D/G)(E/K)(K/N)AQQ(R/H)(R/C)KQ-393]; 48 [385-(E/K)(K/N)AQQ(R/H)(R/C)KQWWNESK-399]; and 66 [421-ICK(L/I)NVAVNIEPQIY-435]. *In silico* predicted promiscuous epitopes: Ia [416-G(N/K)F(I/M)WICK(L/I)-424]; Ib [482-KSYD(Q/E)WITR-490]; Ic [497-VLSNKF(I/K)SVKNAEK-510]; IIa [408-YSVKKRLKG(N/K)-417]; and IIb [418-F(I/M)WICK(L/I)NV-426]

*Abbreviations*: *S*, number of polymorphic (segregating) sites; *M*, total number of mutations; *H*, number of haplotypes; *Hd*, haplotype diversity; π, observed average pairwise nucleotide diversity; *d*_N_, rate of non-synonymous mutations; *d*_S_, rate of synonymous mutations; B, B-cell epitope; T, T-cell epitope; SD, standard deviation

*P < 0.05

## Discussion

Polymorphic antigens with the presence of variant haplotypes in different endemic areas complicate the development of an effective malaria vaccine. PvDBP is an essential ligand involved in the invasion of RBCs by the *P. vivax* parasites, and it appears to have similar drawbacks. Assessment of the level of diversity in the *pvdbp*-II region involved in binding to the Duffy antigen within and between populations helps guide the development of a PvDBP-based vaccine. In this study, we determined the genetic diversity and molecular evolution of *pvdbp* domain II in two Myanmar border populations and compared them with other parasite populations in the GMS and elsewhere in the world. Haplotype diversity (*H*d) from the eastern (0.850) and western (0.930) Myanmar populations were at the similar level or slightly lower than other endemic areas such as PNG (0.936), Brazil (0.934), Colombia (0.985), India (0.921), Thailand (0.983) and Iran (0.944) [17, 27–29, 34, 35, 37]. In addition, the two highest peaks of nucleotide diversity within the *pvdbp*-II sequences in the Myanmar border parasite isolates were localized between Cysteine 5 and 7 of PvDBP-II, which was consistent with several previous reports [28, 30, 31, 47].

The D384G mutation is fixed in the eastern Myanmar border population and reached 92.68% in the western Myanmar isolates. D384G is also highly prevalent in isolates from central Myanmar (85.2%), Thailand (76.7%), Sri Lanka (94%), Iran (61.3%), and Brazil (85%), but it is not prevalent in PNG and Colombia [17, 27–29, 34, 35, 37]. Although eastern and western Myanmar isolates had similar amino acid changes with populations from central Myanmar and Thailand, some mutations found in the Thai isolates (S351C, I367T, S398T, T404R, Q433K and R436T) and central Myanmar isolates (I310L, K386Q, R391H, K455I, K473R, P475A, C477G, Q486E, R490K, D528G, V533M, K541T and A545V) were not identified in either eastern or western Myanmar isolates [3, 29]. It has been reported that amino acids 417, 437 and 503 are responsible for forming a discontinuous epitope of PvDBP-II, which was also the main target of inhibitory antibodies [48, 49]. In this regard, polymorphisms at these residues, either single or combined, could affect the binding efficiency of inhibitory antibodies to PvDBP and help the parasites to evade host immune attacks. All these three mutations were found in eastern and western Myanmar isolates. The N417K mutation was dominant with high frequencies in both eastern (63.5%) and western (87.8%) Myanmar, whereas the W437R mutation was found considerably high in the eastern (62%) but low (2%) in western Myanmar. The I503K variant was present at a similar prevalence in the eastern (47%) and western Myanmar (49%) populations. All these mutations suggest that antibody responses against PvDBP in these regions might be compromised.

The *pvdbp*-II has been found to be subject to strong selection in previous studies [17, 27–29, 34, 35, 37]. Although all neutrality tests and the *d*_N_/*d*_S_ test did not identify significant deviation from neutral in the overall *pvdbp*-II region in the Myanmar parasite populations, sliding window analyses of Fu and Li’s F* test and Tajima’s *D* test did identify a region of *pvdbp*-II gene that might be under positive selection (e.g., codons 372–388). These results were further supported by the high degrees of polymorphism in the B- and T-cell epitopes in the global *P. vivax* populations [26]. In particular, high-level genetic diversity and positive selection were identified in peptide Ia, predicted to be exposed on the surface of the PvDBP protein [26]. Significant positive values observed for the neutrality tests for the codons 363–399 and codons 370–392 regions of *pvdbp*-II suggest a decrease in population size. This appears to be consistent with the scaling up of malaria control in these endemic areas, which has resulted in substantial declines of malaria incidence in recent years. However, the region of codons 542–553 did not follow the same trends with the Fu and Li’s D* test, which showed a significant negative value for this domain, suggesting an excess of singletons, probably generated by particular selective sweeps. Thereby, the codons 542–553 region of *pvdbp*-II may be necessary for maintaining the binding ability of PvDBP-II to DARC. Taken together, the current study supports the theory that strong balancing selection probably imposed by host immunity may have occurred in the *pvdbp*-II region in the GMS parasite populations.

The high value of the recombination parameters observed in global populations suggested that meiotic recombination might have occurred in PvDBP-II. Recombination contributes to genetic diversity of *P. vivax* and may also be the cause for the variation of *pvdbp*-II as reported in several previous studies [26, 50]. The existence of recombination events and the decline of LD index R^2^ with increasing distance between nucleotide sites support that meiotic recombination plays a role in generating diversity in *pvdbp*-II among the Myanmar parasite isolates. This result further corroborated previous reports from other *P. vivax* endemic regions [3, 17, 26–28, 36]. The relatively lower number of the recombination events observed in the eastern Myanmar border isolates might be correlated with decreased *P. vivax* transmission intensity as this region is moving towards malaria elimination.

The *F*_ST_ index was used to access population differentiation due to genetic structure, and *F*_ST_ values over 0.25 generally refer to highly differentiated populations [51]. Whereas major population differentiation was observed between geographically well-separated parasite populations (e.g. GMS *vs* Colombia, GMS *vs* PNG), little population differentiation was observed in parasite from the GMS, as evidenced by the low *F*_ST_ values. This may reflect extensive gene flow among these GMS parasite populations in the recent past, which is further boosted by intensive human migration between these GMS countries, especially in the border regions [2]. Both STRUCTURE and network analyses further confirmed the close relationships among the four GMS populations. The PvDBP-II haplotypes were grouped into four closely related clusters and are shared by all sites.

Genetic diversity of vaccine candidate antigens usually causes poor clinical efficacy of malaria vaccines [52, 53]. Thus, an effective PvDBP-II vaccine should include alleles that induce host immune responses that are sufficiently broad to cover the existing antigenic diversity. However, because of higher genetic diversity of *P. vivax* compared to *P. falciparum*, generating a broad cross-reactive immune response against highly polymorphic asexual stage antigens faces even greater challenges [54]. Our observation that only 15% of PvDBP-II haplotypes were shared among the four populations from the GMS suggests that antigenic diversity will need to be taken into account for a PvDBP-based vaccine. In addition, the Sal I PvDBP-II haplotype was restricted to western and central Myanmar populations at a low frequency (4.76%), indicating that a PvDBP-II vaccine designed based on this reference strain may not work in the GMS. It is noteworthy that the GMS parasite populations did show highly prevalent haplotypes (e.g. H25 at 17.14%), which may serve as the starting point for vaccine development.

## Conclusions

This study shows that *pvdbp* gene is genetically diverse in both the eastern and western Myanmar *P. vivax* isolates, which is comparable to global *P. vivax* populations. Part of the *pvdbp*-II domain is under positive selection, while multiple recombination events further favor diversity. Little genetic differentiation identified among the four populations in the GMS suggests extensive gene flow between these areas. These findings provide important information for understanding the *P. vivax* population structure and its evolution in endemic areas of the GMS, and allow investigators to select dominant haplotypes for designing an effective blood-stage vaccine.

## Data Availability

The datasets supporting the conclusions of this article are included within the article. The *pvdbp*-II sequences of field isolates obtained in this study were submitted to the GenBank database under the accession numbers MN233407-MN233488 for the western Myanmar parasites and MN233489-MN233573 for the eastern Myanmar parasites.

## Abbreviations

RBC: red blood cell
DARC: Duffy antigen receptor for chemokines
PvDBP-II: *Plasmodium vivax* Duffy-binding protein region II
Sal I: Salvador I
*F*_ST_: fixation index
LD: linkage disequilibrium
Rm: recombination event
*d*_N_: mean number of non-synonymous substitutions per non-synonymous site
*d*_S_: mean number of synonymous substitutions per synonymous site
PNG: Papua New Guinea
MCMC: Markov Chain Monte Carlo
SNP: single nucleotide polymorphism
